# 4-(4-Chloro­phen­yl)-8-methyl-2-oxo-1,2,3,4,4a,5,6,7-octa­hydro­quinoline-3-carbonitrile

**DOI:** 10.1107/S1600536811036154

**Published:** 2011-09-14

**Authors:** Abdullah M. Asiri, Abdulrahman O. Al-Youbi, Hassan M. Faidallah, Khadija O. Badahdah, Seik Weng Ng

**Affiliations:** a Chemistry Department, Faculty of Science, King Abdulaziz University, PO Box 80203 Jeddah, Saudi Arabia; b Center of Excellence for Advanced Materials Research, King Abdulaziz University, PO Box 80203 Jeddah, Saudi Arabia; c Department of Chemistry, University of Malaya, 50603 Kuala Lumpur, Malaysia

## Abstract

The six-membered *N*-heterocyclic ring of title compound, C_17_H_17_ClN_2_O, is fused with a methyl-substituted cyclo­hexene ring. The nitro­gen-bearing ring has an envelope conformation with the benzene ring-bearing C atom lying 0.432 (6) Å out of the plane defined by the other five atoms (r.m.s. deviation 0.011 Å); its benzene substituent is aligned at 84.7 (1)° to the latter plane. The cyclo­hexene ring adopts a half-chair conformation. In the crystal, two mol­ecules are linked about a center of inversion by pairs of N–H⋯O hydrogen bonds, generating dimers. An ethyl­ene portion is disordered over two orientations in a 1:1 ratio. The crystal studied was a non-merohedral twin with a 15.3 (1)% minor component.

## Related literature

For a similar compound that has two more H atoms, see: Asiri *et al.* (2011 ▶).
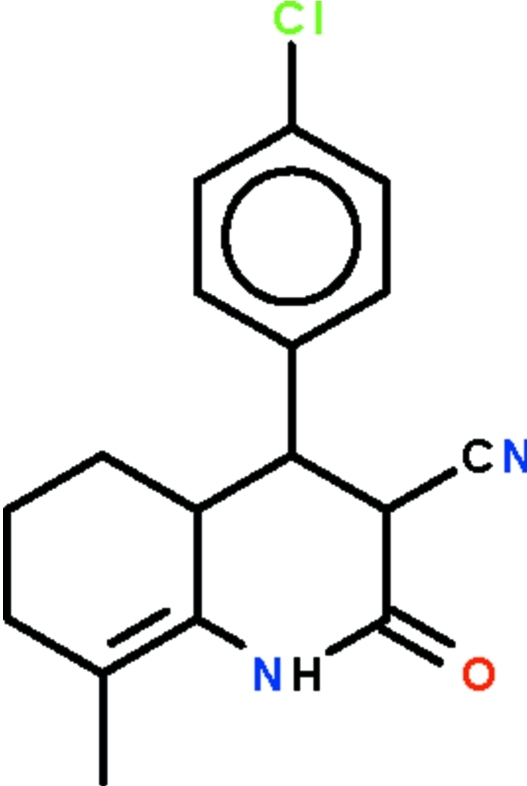


## Experimental

### 

#### Crystal data

C_17_H_17_ClN_2_O
*M* *_r_* = 300.78
Monoclinic, 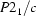

*a* = 11.0699 (7) Å
*b* = 7.6018 (3) Å
*c* = 18.2247 (9) Å
β = 100.505 (6)°
*V* = 1507.92 (13) Å^3^
*Z* = 4
Cu *K*α radiation
μ = 2.24 mm^−1^
*T* = 100 K
0.30 × 0.20 × 0.05 mm

#### Data collection

Agilent SuperNova Dual diffractometer with Atlas detector
Absorption correction: multi-scan (*CrysAlis PRO*; Agilent, 2010 ▶) *T*_min_ = 0.554, *T*_max_ = 0.896
25955 measured reflections
6143 independent reflections
3143 reflections with *I* > 2σ(*I*)
*R*_int_ = 0.020

#### Refinement

*R*[*F* ^2^ > 2σ(*F* ^2^)] = 0.079
*wR*(*F* ^2^) = 0.273
*S* = 1.10
6140 reflections
199 parameters
18 restraints
H-atom parameters constrained
Δρ_max_ = 0.68 e Å^−3^
Δρ_min_ = −0.46 e Å^−3^

### 

Data collection: *CrysAlis PRO* (Agilent, 2010 ▶); cell refinement: *CrysAlis PRO*; data reduction: *CrysAlis PRO*; program(s) used to solve structure: *SHELXS97* (Sheldrick, 2008 ▶); program(s) used to refine structure: *SHELXL97* (Sheldrick, 2008 ▶); molecular graphics: *X-SEED* (Barbour, 2001 ▶); software used to prepare material for publication: *publCIF* (Westrip, 2010 ▶).

## Supplementary Material

Crystal structure: contains datablock(s) global, I. DOI: 10.1107/S1600536811036154/xu5321sup1.cif

Structure factors: contains datablock(s) I. DOI: 10.1107/S1600536811036154/xu5321Isup2.hkl

Supplementary material file. DOI: 10.1107/S1600536811036154/xu5321Isup3.cml

Additional supplementary materials:  crystallographic information; 3D view; checkCIF report

## Figures and Tables

**Table 1:** Hydrogen-bond geometry (Å, °)

*D*—H⋯*A*	*D*—H	H⋯*A*	*D*⋯*A*	*D*—H⋯*A*
N1—H1⋯O1^i^	0.88	2.07	2.923 (3)	162

Symmetry code: (i) 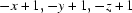.

**Fig. 1.**
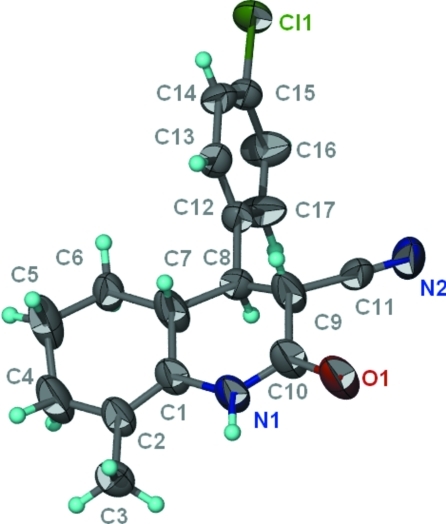
Thermal ellipsoid plot (Barbour, 2001) of C17H17ClN2O at the 70% probability level; hydrogen atoms are drawn as spheres of arbitrary radius. The disorder is not shown.

## supplementary crystallographic information

### Comment

The compound (Scheme I) is a side product that was isolated along with 4-(4-chlorophenyl)-8-methyl-2-oxo-1,2,5,6,7,8-hexahydroquinoline-3- carbonitrile, the expected product (Asiri *et al.*, 2011); the proportion of the two compounds is unknown. The compound has one set of carbon-carbon double-bonds whereas the expected product has two, *i.e.*, it has two more hydrogen atoms. Futhermore, the double-bond is now located in the methyl-substituted cyclohexene ring. The six-membered ring bearing an N atom that is fused with the cyclohexene ring. The nitrogen-bearing ring has the benzene-bearing C atom lying 0.432 (6) Å out of the plane defined by the other five atoms (r.m.s. deviation 0.011 Å); its benzene substituent is aligned at 84.7 (1) °. The cyclohexene ring adopts a half-chair conformation; an ethylene portion is disordered over two positions in a 1:1 ratio (Fig. 1). Two molecules are linked about a center-of-inversion by an N–H···O hydrogen bond to generate a dimer (Table 1).

### Experimental

4-Chlorobenzaldehyde (1.4 g, 10 mmol), 2-methylcyclohexanone (1.2 g, 10 mmol), ethyl cyanoacetate (1.1 g, 10 mmol) and ammonium acetate (6.2 g, 80 mmol) were heated in ethanol (50 ml) for 6 h. The solid product was collected, washed with water and then recrystallized from ethanol.

One crystal was characterized as 4-(4-chlorophenyl)-8-methyl-2-oxo-1,2,5,6,7,8-hexahydroquinoline-3- carbonitrile, the expected product (Asiri *et al.*, 2011). The second type of crystal is not aromatic and it has two extra hydrogen atoms in the nitrogen-bearing ring (Scheme I).

### Refinement

Carbon- and nitrogen-bound H-atoms were placed in calculated positions [C–H 0.95–0.99 and N–H 0.88 Å; *U*_iso_(H) 1.2–1.5 *U*_eq_(C,N)] and were included in the refinement in the riding model approximation. The methyl group was treated as an idealized disordered group with the two positions rotated by 60°.

An ethylene section of the cyclohexene ring is disordered over two positions; the disorder could not be refined, and was regarded as a 1:1 type of disorder. Within the ring, the 1,2-related bond distances were restrained to 1.54±0.01 Å and the 1,3-related non-bonded distances to 2.51±0.01 Å. The temperature factors of the primed atoms were set to those of the umprimed ones; the anisotropic temperature factors were restrained to be nearly isotropic.

Omitted from the refinement owing to bad disagreement were (-2 1 0), (2 3 - 3) and (-4 1 1).

The somewhat large weighting scheme is attributed to the twinned nature of the crystal, and this is compounded by disorder. The minor component refined to 15.3 (1)%.

### Crystal data

**Table:**

C_17_H_17_ClN_2_O	*F*(000) = 632
*M**_r_* = 300.78	*D*_x_ = 1.325 Mg m^−^^3^
Monoclinic, *P*2_1_/*c*	Cu *K*α radiation, λ = 1.54184 Å
Hall symbol: -P 2ybc	Cell parameters from 4615 reflections
*a* = 11.0699 (7) Å	θ = 4.1–74.2°
*b* = 7.6018 (3) Å	µ = 2.24 mm^−^^1^
*c* = 18.2247 (9) Å	*T* = 100 K
β = 100.505 (6)°	Prism, colorless
*V* = 1507.92 (13) Å^3^	0.30 × 0.20 × 0.05 mm
*Z* = 4	

### Data collection

**Table:**

Agilent SuperNova Dual diffractometer with Atlas detector	6143 independent reflections
Radiation source: SuperNova (Cu) X-ray Source	3143 reflections with *I* > 2σ(*I*)
Mirror	*R*_int_ = 0.020
Detector resolution: 10.4041 pixels mm^-1^	θ_max_ = 74.6°, θ_min_ = 4.1°
ω scan	*h* = −13→13
Absorption correction: multi-scan (*CrysAlis PRO*; Agilent, 2010)	*k* = −8→9
*T*_min_ = 0.554, *T*_max_ = 0.896	*l* = −22→22
25955 measured reflections	

### Refinement

**Table:**

Refinement on *F*^2^	Secondary atom site location: difference Fourier map
Least-squares matrix: full	Hydrogen site location: inferred from neighbouring sites
*R*[*F*^2^ > 2σ(*F*^2^)] = 0.079	H-atom parameters constrained
*wR*(*F*^2^) = 0.273	*w* = 1/[σ^2^(*F*_o_^2^) + (0.1311*P*)^2^] where *P* = (*F*_o_^2^ + 2*F*_c_^2^)/3
*S* = 1.10	(Δ/σ)_max_ = 0.001
6140 reflections	Δρ_max_ = 0.68 e Å^−^^3^
199 parameters	Δρ_min_ = −0.46 e Å^−^^3^
18 restraints	Extinction correction: *SHELXL*, Fc^*^=kFc[1+0.001xFc^2^λ^3^/sin(2θ)]^-1/4^
Primary atom site location: structure-invariant direct methods	Extinction coefficient: 0.0036 (12)

### Fractional atomic coordinates and isotropic or equivalent isotropic displacement parameters (Å^2^)

**Table:**

	*x*	*y*	*z*	*U*_iso_*/*U*_eq_	Occ. (<1)
Cl1	0.94318 (9)	0.09540 (11)	0.08219 (4)	0.0756 (3)	
O1	0.5121 (3)	0.5375 (4)	0.41298 (14)	0.1111 (12)	
N1	0.6012 (3)	0.2957 (3)	0.47183 (14)	0.0731 (8)	
H1	0.5690	0.3234	0.5111	0.088*	
N2	0.6458 (5)	0.6373 (4)	0.2684 (2)	0.1182 (15)	
C1	0.6727 (3)	0.1423 (4)	0.47779 (17)	0.0667 (9)	
C2	0.6849 (3)	0.0426 (4)	0.53982 (18)	0.0724 (9)	
C3	0.6222 (4)	0.0736 (5)	0.6033 (2)	0.0845 (11)	
H3A	0.6609	0.0020	0.6459	0.127*	0.50
H3B	0.5354	0.0410	0.5892	0.127*	0.50
H3C	0.6286	0.1983	0.6171	0.127*	0.50
H3D	0.5557	0.1589	0.5889	0.127*	0.50
H3E	0.6812	0.1198	0.6456	0.127*	0.50
H3F	0.5880	−0.0374	0.6177	0.127*	0.50
C4	0.7734 (15)	−0.1120 (17)	0.5423 (7)	0.102 (3)	0.50
H4A	0.7516	−0.2009	0.5774	0.123*	0.50
H4B	0.8573	−0.0698	0.5628	0.123*	0.50
C5	0.7757 (9)	−0.2041 (13)	0.4660 (5)	0.084 (3)	0.50
H5A	0.8353	−0.3025	0.4710	0.101*	0.50
H5B	0.6934	−0.2466	0.4420	0.101*	0.50
C6	0.8189 (4)	−0.0437 (5)	0.4237 (2)	0.1017 (15)	
H6A	0.8961	0.0038	0.4531	0.122*	0.50
H6B	0.8364	−0.0839	0.3750	0.122*	0.50
H6C	0.8999	0.0116	0.4236	0.122*	0.50
H6D	0.8025	−0.1232	0.3800	0.122*	0.50
C4'	0.7531 (16)	−0.1306 (17)	0.5520 (8)	0.102 (3)	0.50
H4'A	0.6935	−0.2284	0.5498	0.123*	0.50
H4'B	0.8061	−0.1314	0.6020	0.123*	0.50
C5'	0.8316 (9)	−0.1565 (13)	0.4924 (4)	0.084 (3)	0.50
H5'A	0.9183	−0.1473	0.5179	0.101*	0.50
H5'B	0.8190	−0.2796	0.4749	0.101*	0.50
C7	0.7272 (4)	0.0954 (5)	0.4112 (2)	0.0914 (13)	
H7	0.6568	0.0294	0.3813	0.110*	
C8	0.7367 (3)	0.2397 (4)	0.36058 (16)	0.0606 (7)	
H8	0.8027	0.3136	0.3905	0.073*	
C9	0.6351 (4)	0.3604 (5)	0.3464 (2)	0.0936 (14)	
H9	0.5698	0.2916	0.3134	0.112*	
C10	0.5762 (4)	0.4046 (5)	0.41368 (19)	0.0866 (12)	
C11	0.6450 (4)	0.5194 (4)	0.30371 (18)	0.0706 (9)	
C12	0.7915 (3)	0.1957 (3)	0.29240 (15)	0.0555 (7)	
C13	0.7323 (3)	0.0835 (4)	0.23823 (16)	0.0603 (7)	
H13	0.6580	0.0285	0.2449	0.072*	
C14	0.7794 (3)	0.0495 (4)	0.17398 (17)	0.0645 (8)	
H14	0.7382	−0.0293	0.1373	0.077*	
C15	0.8864 (3)	0.1311 (4)	0.16408 (16)	0.0588 (7)	
C16	0.9464 (4)	0.2382 (5)	0.2169 (2)	0.0898 (12)	
H16	1.0208	0.2924	0.2100	0.108*	
C17	0.9001 (3)	0.2709 (5)	0.28208 (19)	0.0845 (11)	
H17	0.9440	0.3456	0.3195	0.101*	

### Atomic displacement parameters (Å^2^)

**Table:**

	*U*^11^	*U*^22^	*U*^33^	*U*^12^	*U*^13^	*U*^23^
Cl1	0.1037 (7)	0.0772 (5)	0.0564 (4)	0.0074 (4)	0.0422 (4)	0.0022 (4)
O1	0.156 (3)	0.115 (2)	0.0785 (17)	0.082 (2)	0.0643 (18)	0.0375 (15)
N1	0.0919 (19)	0.0772 (17)	0.0608 (14)	0.0330 (15)	0.0417 (14)	0.0202 (13)
N2	0.222 (5)	0.0612 (18)	0.094 (2)	0.028 (2)	0.088 (3)	0.0162 (17)
C1	0.073 (2)	0.0755 (19)	0.0606 (17)	0.0233 (16)	0.0353 (15)	0.0164 (15)
C2	0.081 (2)	0.076 (2)	0.0669 (19)	0.0181 (17)	0.0324 (17)	0.0249 (16)
C3	0.116 (3)	0.082 (2)	0.0614 (19)	−0.009 (2)	0.031 (2)	0.0071 (17)
C4	0.140 (5)	0.105 (4)	0.083 (4)	0.046 (4)	0.074 (4)	0.058 (3)
C5	0.098 (6)	0.087 (5)	0.073 (5)	0.043 (4)	0.035 (4)	0.032 (4)
C6	0.137 (4)	0.097 (3)	0.089 (3)	0.064 (3)	0.066 (3)	0.042 (2)
C4'	0.140 (5)	0.105 (4)	0.083 (4)	0.046 (4)	0.074 (4)	0.058 (3)
C5'	0.098 (6)	0.087 (5)	0.073 (5)	0.043 (4)	0.035 (4)	0.032 (4)
C7	0.117 (3)	0.095 (3)	0.079 (2)	0.050 (2)	0.062 (2)	0.039 (2)
C8	0.082 (2)	0.0543 (15)	0.0518 (14)	0.0011 (15)	0.0290 (15)	0.0014 (12)
C9	0.138 (3)	0.085 (2)	0.074 (2)	0.054 (2)	0.061 (2)	0.0329 (19)
C10	0.112 (3)	0.090 (2)	0.069 (2)	0.051 (2)	0.049 (2)	0.0215 (18)
C11	0.112 (3)	0.0497 (15)	0.0594 (17)	0.0056 (17)	0.0401 (18)	−0.0032 (14)
C12	0.0720 (19)	0.0486 (14)	0.0511 (14)	0.0042 (13)	0.0253 (13)	0.0023 (11)
C13	0.0702 (18)	0.0533 (15)	0.0645 (17)	−0.0016 (14)	0.0308 (15)	0.0010 (13)
C14	0.083 (2)	0.0580 (17)	0.0565 (16)	−0.0004 (15)	0.0243 (16)	−0.0096 (13)
C15	0.0743 (19)	0.0574 (16)	0.0511 (15)	0.0018 (14)	0.0284 (14)	0.0009 (12)
C16	0.099 (3)	0.108 (3)	0.075 (2)	−0.038 (2)	0.049 (2)	−0.019 (2)
C17	0.093 (2)	0.103 (3)	0.0655 (19)	−0.038 (2)	0.0371 (19)	−0.0272 (19)

### Geometric parameters (Å, °)

**Table:**

Cl1—C15	1.743 (3)	C6—H6B	0.9900
O1—C10	1.234 (4)	C6—H6C	0.9900
N1—C10	1.333 (4)	C6—H6D	0.9900
N1—C1	1.403 (4)	C4'—C5'	1.523 (9)
N1—H1	0.8800	C4'—H4'A	0.9900
N2—C11	1.104 (4)	C4'—H4'B	0.9900
C1—C2	1.347 (4)	C5'—H5'A	0.9900
C1—C7	1.494 (4)	C5'—H5'B	0.9900
C2—C3	1.474 (4)	C7—C8	1.449 (4)
C2—C4'	1.513 (8)	C7—H7	1.0000
C2—C4	1.525 (8)	C8—C9	1.438 (4)
C3—H3A	0.9800	C8—C12	1.516 (3)
C3—H3B	0.9800	C8—H8	1.0000
C3—H3C	0.9800	C9—C11	1.452 (4)
C3—H3D	0.9800	C9—C10	1.527 (4)
C3—H3E	0.9800	C9—H9	1.0000
C3—H3F	0.9800	C12—C17	1.375 (4)
C4—C5	1.560 (9)	C12—C13	1.376 (4)
C4—H4A	0.9900	C13—C14	1.390 (4)
C4—H4B	0.9900	C13—H13	0.9500
C5—C6	1.564 (10)	C14—C15	1.378 (4)
C5—H5A	0.9900	C14—H14	0.9500
C5—H5B	0.9900	C15—C16	1.341 (5)
C6—C7	1.455 (4)	C16—C17	1.399 (4)
C6—C5'	1.502 (6)	C16—H16	0.9500
C6—H6A	0.9900	C17—H17	0.9500
			
C10—N1—C1	127.6 (2)	C2—C4'—H4'B	109.7
C10—N1—H1	116.2	C5'—C4'—H4'B	109.7
C1—N1—H1	116.2	H4'A—C4'—H4'B	108.2
C2—C1—N1	120.1 (3)	C6—C5'—C4'	122.8 (8)
C2—C1—C7	123.6 (3)	C6—C5'—H5'A	106.6
N1—C1—C7	116.2 (2)	C4'—C5'—H5'A	106.6
C1—C2—C3	125.4 (3)	C6—C5'—H5'B	106.6
C1—C2—C4'	125.7 (5)	C4'—C5'—H5'B	106.6
C3—C2—C4'	108.5 (5)	H5'A—C5'—H5'B	106.6
C1—C2—C4	115.1 (5)	C8—C7—C6	121.4 (3)
C3—C2—C4	119.5 (5)	C8—C7—C1	115.0 (3)
C2—C3—H3A	109.5	C6—C7—C1	114.8 (3)
C2—C3—H3B	109.5	C8—C7—H7	100.0
H3A—C3—H3B	109.5	C6—C7—H7	100.0
C2—C3—H3C	109.5	C1—C7—H7	100.0
H3A—C3—H3C	109.5	C9—C8—C7	116.9 (3)
H3B—C3—H3C	109.5	C9—C8—C12	114.2 (3)
C2—C3—H3D	109.5	C7—C8—C12	116.1 (2)
C2—C3—H3E	109.5	C9—C8—H8	102.1
H3D—C3—H3E	109.5	C7—C8—H8	102.1
C2—C3—H3F	109.5	C12—C8—H8	102.1
H3D—C3—H3F	109.5	C8—C9—C11	119.6 (3)
H3E—C3—H3F	109.5	C8—C9—C10	115.5 (3)
C2—C4—C5	115.8 (9)	C11—C9—C10	109.4 (3)
C2—C4—H4A	108.3	C8—C9—H9	103.3
C5—C4—H4A	108.3	C11—C9—H9	103.3
C2—C4—H4B	108.3	C10—C9—H9	103.3
C5—C4—H4B	108.3	O1—C10—N1	123.5 (3)
H4A—C4—H4B	107.4	O1—C10—C9	120.1 (3)
C6—C5—C4	98.4 (8)	N1—C10—C9	116.4 (3)
C6—C5—H5A	112.1	N2—C11—C9	175.5 (5)
C4—C5—H5A	112.1	C17—C12—C13	118.2 (3)
C6—C5—H5B	112.1	C17—C12—C8	120.7 (3)
C4—C5—H5B	112.1	C13—C12—C8	121.0 (3)
H5A—C5—H5B	109.7	C12—C13—C14	121.1 (3)
C7—C6—C5'	120.1 (5)	C12—C13—H13	119.4
C7—C6—C5	112.1 (5)	C14—C13—H13	119.4
C7—C6—H6A	109.2	C15—C14—C13	119.3 (3)
C5—C6—H6A	109.2	C15—C14—H14	120.3
C7—C6—H6B	109.2	C13—C14—H14	120.3
C5—C6—H6B	109.2	C16—C15—C14	120.3 (3)
H6A—C6—H6B	107.9	C16—C15—Cl1	120.0 (2)
C7—C6—H6C	107.3	C14—C15—Cl1	119.7 (2)
C5'—C6—H6C	107.3	C15—C16—C17	120.5 (3)
C7—C6—H6D	107.3	C15—C16—H16	119.8
C5'—C6—H6D	107.3	C17—C16—H16	119.8
H6C—C6—H6D	106.9	C12—C17—C16	120.4 (3)
C2—C4'—C5'	109.9 (9)	C12—C17—H17	119.8
C2—C4'—H4'A	109.7	C16—C17—H17	119.8
C5'—C4'—H4'A	109.7		
			
C10—N1—C1—C2	−177.3 (4)	C6—C7—C8—C9	173.9 (4)
C10—N1—C1—C7	0.0 (6)	C1—C7—C8—C9	−40.3 (6)
N1—C1—C2—C3	4.0 (6)	C6—C7—C8—C12	34.3 (6)
C7—C1—C2—C3	−173.1 (4)	C1—C7—C8—C12	−179.9 (3)
N1—C1—C2—C4'	176.4 (10)	C7—C8—C9—C11	172.5 (4)
C7—C1—C2—C4'	−0.6 (12)	C12—C8—C9—C11	−47.2 (5)
N1—C1—C2—C4	−175.1 (8)	C7—C8—C9—C10	38.5 (6)
C7—C1—C2—C4	7.8 (10)	C12—C8—C9—C10	178.8 (3)
C1—C2—C4—C5	−35.6 (14)	C1—N1—C10—O1	−180.0 (4)
C3—C2—C4—C5	145.3 (8)	C1—N1—C10—C9	−1.9 (6)
C4'—C2—C4—C5	112 (6)	C8—C9—C10—O1	161.0 (4)
C2—C4—C5—C6	61.4 (12)	C11—C9—C10—O1	22.5 (6)
C4—C5—C6—C7	−66.5 (8)	C8—C9—C10—N1	−17.2 (6)
C4—C5—C6—C5'	45.8 (11)	C11—C9—C10—N1	−155.6 (4)
C1—C2—C4'—C5'	13.3 (18)	C9—C8—C12—C17	102.9 (4)
C3—C2—C4'—C5'	−173.2 (10)	C7—C8—C12—C17	−116.5 (4)
C4—C2—C4'—C5'	−23 (5)	C9—C8—C12—C13	−75.2 (4)
C7—C6—C5'—C4'	−1.2 (15)	C7—C8—C12—C13	65.4 (4)
C5—C6—C5'—C4'	−83.5 (18)	C17—C12—C13—C14	−1.2 (5)
C2—C4'—C5'—C6	−12.1 (19)	C8—C12—C13—C14	176.9 (3)
C5'—C6—C7—C8	160.1 (6)	C12—C13—C14—C15	−0.8 (5)
C5—C6—C7—C8	−167.6 (5)	C13—C14—C15—C16	2.0 (5)
C5'—C6—C7—C1	14.2 (8)	C13—C14—C15—Cl1	−177.7 (2)
C5—C6—C7—C1	46.6 (7)	C14—C15—C16—C17	−1.1 (6)
C2—C1—C7—C8	−162.0 (4)	Cl1—C15—C16—C17	178.6 (3)
N1—C1—C7—C8	20.8 (5)	C13—C12—C17—C16	2.1 (6)
C2—C1—C7—C6	−14.0 (6)	C8—C12—C17—C16	−176.0 (4)
N1—C1—C7—C6	168.9 (4)	C15—C16—C17—C12	−1.0 (7)

### Hydrogen-bond geometry (Å, °)

**Table:**

*D*—H···*A*	*D*—H	H···*A*	*D*···*A*	*D*—H···*A*
N1—H1···O1^i^	0.88	2.07	2.923 (3)	162

Symmetry codes: (i) −*x*+1, −*y*+1, −*z*+1.
